# Functionally Flexible Signaling and the Origin of Language

**DOI:** 10.3389/fpsyg.2020.626138

**Published:** 2021-01-26

**Authors:** D. Kimbrough Oller, Ulrike Griebel

**Affiliations:** ^1^School of Communication Sciences and Disorders, The University of Memphis, Memphis, TN, United States; ^2^Institute for Intelligent Systems, The University of Memphis, Memphis, TN, United States; ^3^Konrad Lorenz Institute for Evolution and Cognition Research, Klosterneuburg, Austria

**Keywords:** vocal development, honest signaling, origin of language, evolution of language, babbling, vocal learning, comparative psychology

## Abstract

At the earliest break of ancient hominins from their primate relatives in vocal communication, we propose a selection pressure on vocal fitness signaling by hominin infants. Exploratory vocalizations, not tied to expression of distress or immediate need, could have helped persuade parents of the wellness and viability of the infants who produced them. We hypothesize that hominin parents invested more in infants who produced such signals of fitness plentifully, neglecting or abandoning them less often than infants who produced the sounds less frequently. Selection for such exploratory vocalization provided a critically important inclination and capability relevant to language, we reason, because the system that encouraged spontaneous vocalization also made vocalization functionally flexible to an extent that has not been observed in any other animal. Although this vocal flexibility did not by itself create language, it provided an essential foundation upon which language would evolve through a variety of additional steps. In evaluating this speculation, we consider presumable barriers to evolving language that are thought to be implications of Darwinian Theory. It has been claimed that communication always involves sender self-interest and that self-interest leads to deceit, which is countered through clever detection by receivers. The constant battle of senders and receivers has been thought to pose an insuperable challenge to honest communication, which has been viewed as a requirement of language. To make communication honest, it has been proposed that stable signaling requires costly handicaps for the sender, and since language cannot entail high cost, the reasoning has suggested an insurmountable obstacle to the evolution of language. We think this presumed honesty barrier is an illusion that can be revealed by recognition of the fact that language is not inherently honest and in light of the distinction between illocutionary force and semantics. Our paper also considers barriers to the evolution of language (not having to do with honesty) that we think may have actually played important roles in preventing species other than humans from evolving language.

## Overview

A key goal of our research is to discover the most fundamental vocal capabilities and inclinations upon which language was founded, long before the first word was spoken. Further, we seek to posit evolutionary pressures that may have selected for these capabilities, a task that requires positing advantages, which could not have involved the advantages of language, which did not exist at that point. An initial step that may have moved hominins beyond the primate communicative background is *vocal functional flexibility* (VFF). We have long argued that the natural laboratory of human vocal development provides key evidence relevant to the search for origins of language (Oller, 2000; Griebel and Oller, 2008). VFF is seen from the first month of life in human infant “protophones” (Papaeliou et al., 2002; Scheiner et al., 2002; Oller et al., 2013; Jhang and Oller, 2017), the precursors to speech, including categories termed squeals, vocants (vowel-like sounds), and growls. The protophones serve different functions on different occasions. All protophone types are usually produced playfully or exploratorily with no obvious social intention or social directivity and with neutral facial affect (Long et al., 2020). But the same sounds are also produced on different occasions with positive or negative facial affect, suggesting, for example, exultation or complaint. For example, a squeal sound can be used (1) on one occasion with a big smile portraying apparent exultation, (2) on another occasion with an intense grimace, making an apparent complaint, even suggesting the infant is about to start crying, and (3) on yet another occasion, when the same infant is alone and playing quietly, with a neutral facial expression and no apparent social intent, merely exploring the sound.

Vocal functional flexibility is present throughout human life, since every linguistic signal must be functionally flexible. Any word, for example, must be able to serve a wide variety of different functions (“illocutionary forces,” see below) on different occasions of use. We must even be able to pronounce any word just for the interest of doing it. The two facts (1) that VFF is present from the first month of human life, and (2) that VFF is a foundational requirement of vocal language, suggest that one of the first evolutionary steps that differentiated ancient hominins from their primate relatives in communicative capabilities may have been VFF.

In other primates, vocal flexibility is far more limited because their vocal signals appear to be required to have *particular beneficial effects in the here and now* – later effects are of course possible, but not the focus of the pressures that selected the signals. In this paper, we propose an evolutionary scenario where hominin infant fitness signaling through vocalizations with VFF could have been naturally selected. Importantly these vocalizations would have often had no necessary *immediate* communicatively generated benefits to the infant, just as is the case with modern human protophones. The primary benefits could occur later, when caregivers could invest in infant welfare based on a cumulative conscious or unconscious recollection of the infant fitness signals. The key point is that vocal signals of infant hominins, in this scenario, were selected in a way that left them free of immediate socio-functional requirements. From this platform of infant vocalization and parental awareness of it, we propose that natural selection of infants who showed vocal fitness signaling could have instigated selection of steadily increasing VFF in hominins, thus forming a foundation for and moving them in the direction of language. Subsequent steps built upon the foundation of VFF were, in accord with our proposal, necessary to establish symbolic content in signaling.

Our paper will consider the barrier to the evolution of language that has most often been proposed. The contention is that human language constitutes “honest signaling” (Fitch, 2004) and that because communication is inherently selfish and therefore inclined to deception (Dawkins and Krebs, 1978), language evolution is problematical. In a rebuttal of this line of reasoning, we shall argue that language is not in fact inherently honest, and we shall elucidate this fact by unpacking the distinction between illocutionary force and semantic content in communication (Austin, 1962), a distinction that also helps to illustrate and clarify the nature of functional flexibility. In Appendix A of the Supplementary Material, we offer additional reasons to reject the honest signaling argument. In Appendix B, we supply additional thoughts about a strategy for research on the origin of language along with possible foundations of language that can be seen in evolved communication signals of other species.

## The Critical Nature of Vocal Functional Flexibility in Language

Language is a capability and an inclination that evolved in ancient humans but must be developed within each individual. The emphasis on *inclination* is important because humans use language copiously, imaginatively, and often frivolously, sometimes with no social purpose but just for the pleasure of toying with language itself. In addition, as indicated above, from the first month of life, human infants produce protophones, not bound by any particular emotional state. In fact protophones are produced most commonly in apparent comfort and lack of immediate social goals (Oller et al., 2013; Jhang and Oller, 2017). Even when infants are alone in a room and comfortable, all-day recordings show that protophone production is common, yielding 3–4 utterances per minute (Oller et al., 2019a), and similar rates are observed for infants in the presence of a mother who, for example, is reading silently (Iyer et al., 2016). It is important, however, to emphasize that all the types of protophones that have been recognized as pertaining to the common infant repertoire are also produced in varying states of positive or negative emotion on different occasions, suggesting the protophones can indeed be used to express states with immediate communicative import, e.g., intended to solicit immediate attention from the caregiver.

Counts of protophones based on all-day recordings of infants in their homes show a huge rate, ~5 per minute during wakefulness, ~3,500 per day (Oller et al., 2019a), which is 5–10 times higher than the rate of crying even in the first month. The research shows that protophone production at high rates occurs from as soon as human infants can breathe on their own, as illustrated through all-day recordings of prematurely-born infants still in neonatal intensive care. Evidence of the robustness of the tendency to produce protophones copiously has been observed in American and European infants that have been studied longitudinally for many years (Stark, 1981; Elbers, 1982; Koopmans-van Beinum and van der Stelt, 1986; Stoel-Gammon, 1992), across infants with very different levels of socio-economic status (Eilers et al., 1993, 1997; Oller et al., 1995), across infants with very different languages in the home (Oller and Eilers, 1982; Holmgren et al., 1986; Lee et al., 2017), and even across infants who are later diagnosed with a wide variety of communication disorders (Oller and Eilers, 1988; Vinter, 1994; Masataka, 2001; Patten et al., 2014; Nyman and Lohmander, 2018). This seemingly obsessive human vocal tendency does not subside later in life, with all-day recordings suggesting human adults speaking English produce on the order of 16,000 words per day (Mehl et al., 2007).

There is abundant evidence that this human inclination to speak is endogenous. Consider how often we adults talk to ourselves, sometimes out loud, intending for no other person to hear us, or mutter to limit the possibility that we might be caught at it. But just as important, the infant tendency to produce protophones is not primarily driven by attempts to communicate a particular emotional state (or anything else) to anyone. The great majority of protophones appear to be directed to no one (Long et al., 2020), but seem instead to constitute a kind of exploratory activity, where the infant investigates the nature of the vocal capacity itself and of the types of sounds that can be produced. Even infants born deaf produce massive numbers of protophones, with no evidence that the rate is lower across the first year than in hearing infants (Iyer and Oller, 2008). The conclusion seems inevitable that this vocal activity is pleasurable to infants, pursued in much the same way infants explore objects with their hands, eyes, and mouths, in an apparent attempt to understand the physical world. It is as if the human vocal capacity has come to be engaged for the purpose of playful exploratory activity, similarly to how the hands are engaged with the world in all primates. The vocal exploration yields an understanding of the acoustic properties resulting from infants’ own vocal actions and the relations between those sounds and their kinesthetic accompaniments. This vocal exploratory/seeking behavior seems to be inherently reinforced just as other forms of play or Seeking behavior (see below) are deemed to be inherently pleasurable (Panksepp, 1982; Bekoff and Byers, 1998; Panksepp and Biven, 2012).

Of course there are other animals that produce abundant communicative vocalization. But something critically important for language appears to be absent in vocal activities of non-human apes: There appears to be no tendency to produce vocalizations exploratorily, playfully, seemingly for the sake of the sound experience itself, rather than for the sake of immediate communicative goals (Oller et al., 2019b). Another aspect of this apparent difference is that every human protophone type (by definition, the protophones do not include vegetative sounds or early infant cry or laughter) is produced with VFF, free to be expressed in any state of emotion or intent, whereas non-human vocalizations appear to be much more restricted to being produced as specific (although sometimes mixed) emotional expressions that primarily serve particular functional ends in the here and now.

Also in accord with the principle of VFF in adult humans, no immediate communicative intent (i.e., pursuit of a receiver reaction in the moment) is necessary for any particular language event type to occur, although clearly language would not have evolved had communication with others (both for immediate and long-term effects) not driven the selection of language abilities. We face an apparent paradox. Language is motivated and sustained by communication, but its nature requires that it be possible to use it “non-communicatively” – i.e., playfully and/or exploratorily. If it were not so, the capability would not be truly functionally flexible. So to form a foundation for vocal language, it is necessary for nature to select for a tendency to vocalize without any apparent immediate communicative purpose. Yet that tendency must have significant positive consequences for vocalizers in their own lifetimes. The selection advantage, we propose here depends on caregivers who notice the exploratory sounds of their infants, whether consciously or not, and who use the evidence of wellness inherent in those infant sounds (and the ones that are socially-directed as well) to modulate their investment in the infants’ nurturance.

Empirical tests of the hypothesis that fitness signaling drives protophone production in modern infants can be envisioned in both behavioral and physiological domains. In the behavioral domain, one might predict significant correlations between rate of protophone production across individual infants (perhaps especially the rate of production of protophones when infants are comfortable) and level of parental investment in individual infant welfare. The correlations, we imagine might be most discernible in societies with high infant mortality. Low infant mortality in modern societies appears to have made it possible for many parents to invest most heavily in their least fit infants, in the hopes that all their offspring will be successful – so research in the most informative settings may be difficult to implement. In the physiological domain, one might predict increases in caregiver care-related neurochemicals such as oxytocin when they listen to protophone production. We are planning and encouraging research in both these domains.

We have argued that VFF is a foundation upon which all other aspects of vocal language depend (Oller et al., 2016). The argument is simple and intuitive, relying on the idea that some capabilities are required to develop early in order for others to develop later, because the later ones logically and practically depend on the earlier ones. The argument is supported empirically by the fact that human infants developing language actually go through the steps characterized in the natural logic. The first step in vocal language, as witnessed in longitudinal research, is the exercise of vocalization, copiously, playfully, and with no necessary expressed intent to communicate with others in the short term. This step seems obligatorily to involve VFF, since longitudinal research shows that endogenous, exploratory vocalization is always accompanied by VFF. In addition, without available endogenous infant vocalizations, caregivers would find no raw material with which to engage their infants in vocal interaction (Stern et al., 1975; Jaffe et al., 2001; Gratier et al., 2015), and consequently could not entrain infants in vocal turn-taking (Dominguez et al., 2016). Without vocal turn-taking, infants would not learn to participate in and contribute to protoconversation (Gratier and Devouche, 2011; Yoo et al., 2018). Without infant active participation in protoconversation, using vocalizations with VFF, systematic vocal imitation of new forms would not be possible (Jones, 2009; Long et al., 2019). Without these kinds of foundations, words and sentences could never be developed. This line of reasoning, illustrating that endogenous functionally flexible vocalization forms the initial platform for other critical developments necessary for language, has been presented in detail in other publications cited above, and is consistent with the well-documented facts of infant vocal and early language development summarized with citations in Oller et al. (2016).

Selection pressure on vocal flexibility must have affected hominins much more than closely related species because the functionally flexible capacity and inclination contrasts sharply with vocal inclinations in other apes. So, we are faced with the question: what was different about the situation where hominin vocal capacities must have passed through a phase transition into massively flexible vocal actions, while other apes remained more vocally constrained? The answer, we propose requires us to begin by taking stock of the nature of vocal communication in apes as well as in other non-human primates.

## Emotional Expression and Vocal Communication in Humans and Other Primates

Our current view of the vocal systems of other primates is largely consistent with the original formulation of Darwin (1872), who proposed that vocal actions in many species, including apes and other primates are primarily emotional expressions. These expressions are sometimes complex and are clearly adaptable to circumstances (Snowdon et al., 1997; Crockford and Boesch, 2003; Hopkins et al., 2011), but they are fundamentally emotional nonetheless (Oller et al., 2019b).

The perspective on the role of emotion in communication has been informed recently by the work of Jaak Panksepp, who proposed seven basic emotions in mammals (Panksepp, 2011; Panksepp and Biven, 2012). Panksepp’s perspective is discussed in detail in a separate paper in this volume (Griebel and Oller). Below, capitalized emotion terms are drawn from Panksepp’s seven: Rage, Fear, Lust, Care, Panic (Isolation/Social need), Play (specifically Social Play), and Seeking. The most important point to emphasize here is that one of the seven, the Seeking system, is portrayed as a foundational emotion by which mammals (and presumably other metazoans) are driven to explore their worlds and are inherently rewarded by a sense of pleasure in the exploration itself. Note that the Seeking system can inspire exploratory, playful interaction with conspecifics and thus can activate the Social Play system, but most playful human infant vocalization seems to be independent of sociality, and thus, we propose that protophone production is primarily driven by the Seeking system rather than the Social Play system. A Seeking system is typically not present in other models of emotion (Eckman, 1994), but, we deem it a major advance in our understanding of emotion and of the basis for the massively endogenous and exploratory nature of human vocalization.

Vocalization in primates (except humans) is not explored for its own sake as far as we know, and thus it appears to be dissociated from the Seeking system in non-human primates. Instead, each vocalization type in mature non-human primates tends to be an expression of some other emotional state, selected to serve immediate, here-and-now functions. For example, some vocalization types tend to occur abruptly in response to Fear (distress and alarm calls), some to Rage (threats), some to Panic/Social Need (isolation calls, contact calls, and positive arousal calls), and some to Social Play (laughter). All these vocalization types can occur in circumstances as different as eating, traveling, and grooming, because all the emotional states can occur in any physical circumstance; e.g., in a feeding circumstance, competition for food can elicit the Rage system (possibly yielding vocal threats), the need to calm competitive tendencies regarding food can elicit the Care system (possibly yielding positive arousal/affiliation calls), and or perception of a predator can elicit Fear and/or Rage (possibly yielding a distress/alarm or threat call or a combination of them). We know of no evidence that any vocalization type in primates is confined tightly to any particular circumstance – rather emotions are inspired flexibly by events both internal and external to the organism, and their expression at each point in time may reflect the state of the producer more directly than the state of the environment. Importantly, emotional signals are flexible enough that they can sometimes be inhibited even when the corresponding triggering circumstances occur (Laporte and Zuberbühler, 2010; Owren et al., 2011).

So-called “predator-specific alarm calls” have been acknowledged, even in the earliest publications on the topic, to occur both in the circumstance of perceiving a predator and in intra-specific aggression (Seyfarth et al., 1980), and this point has been reconfirmed and elaborated in more recent revisiting of data regarding the species (the vervet monkey) on which the original alarm call research was done (Price, 2013; Price et al., 2015). Clearly the emotions of Fear and Rage are adaptable to eliciting vocal actions in widely different circumstances. Of course, in this argument, we do not dispute the idea that the physical environment can under some conditions elicit a particular emotion or a corresponding vocalization fairly reliably.

Vocalization in primates sometimes occurs in circumstances of low arousal, and in such cases one might ask if there is any emotion at all involved. Are such vocalizations equivalents to the protophones of human infants, displaying VFF? The answer must of course be determined empirically, and a trustworthy answer will depend on judgments of the functions of vocalizations occurring in their varying contexts. Our own research with three bonobo infants and their mothers in the first year (Oller et al., 2019b) suggests that some of the low arousal bonobo infant sounds, we observed (having occurred less than 1/10 as often as human protophones) were acoustically similar to some protophones, but we saw no evidence of human-like VFF. Essentially all the low arousal bonobo sounds that were produced and could be judged for function appeared to have negative valence (the infant trying to get back to mother and away from a harassing other bonobo, the infant whimpering for help after having climbed up on the cage and seemingly feeling unsure how to get down, and so on). These vocalizations could perhaps be attributed to Fear and/or Panic/Isolation. The judgment of valence in our research was based on how the infant acted before, during, and immediately after the vocalization, other events occurring at the time, and how the mother responded, often by picking the infant up and comforting or feeding him. Notably the bonobo mothers, while being very responsive physically, comforting infants or getting them out of trouble, never in 1,700 min of coded observation, responded to an infant vocalization with a vocalization of their own. Cases of bonobo infant vocalizations judged to have positive valence were deemed to be laughter, and not protophone-like (a laughter event in human infants is not treated as a protophone either). Perhaps most important, there was never a case of a vocalization at any intensity produced by a bonobo infant that was judged to be exploratory or playful – for criteria used in our human infant research to judge exploratory vocalization, see Long et al. (2020). In contrast, human infant protophones are abundantly judged to be exploratory, because they frequently show no sign of being directed to any one, are not judged to be based on discomfort, are not seen to have elicited immediate assistance, and are often produced when infants are alone in a room. At the same time, all the protophones of human infants show VFF and thus do occur on other occasions with social directivity, with signs of discomfort, with signs of delight, or in circumstances that elicit attention (often vocal attention) and/or help.

So far, there has been no convincing demonstration of functional flexibility in vocalizations of non-human primates, although there have been many demonstrations of *contextual* flexibility, that is, demonstrations that the same kind of sound occurs in different physical situations (de Waal, 1982; Harcourt et al., 1993; Biben and Bernhards, 1995; Bermejo and Omedes, 1999; Crockford and Boesch, 2003; Hopkins et al., 2011; Taglialatela et al., 2012). That a particular vocal type can occur in multiple physical situations can, of course, simply imply that similar emotional states occur in different physical situations.

One direct attempt to demonstrate VFF in adult bonobo vocalizations (Clay et al., 2015) did not actually address the issue, for two reasons: First, the study claimed to show that a particular vocal type (the peep) occurred in three situations: during aggression, traveling, and feeding. The authors interpreted the peeps as being negatively valenced during aggression, neutrally valenced during traveling, and positively valenced during feeding. This contextual variability does not, however, actually determine the function or emotional valence of the peeps occurring in these three different contexts. The same emotion that produces a peep could occur during any of the three contexts, in which case the function could be thought of, e.g., as an expression of annoyance (mild Rage) in all three cases or as an expression of Panic/Social Need in all three cases. It is untenable to assume that there exist one-to-one mappings of contexts to functions of vocalizations in primates (as was done in Clay et al., 2015) or of contexts to emotional states, since all emotions can occur in a variety of physical contexts, and correspondingly a variety functions can be served by vocal expression of those emotions in those varying contexts. This kind of flexibility is a defining characteristic of emotions in contrast to reflexes, which are more rigid and show shorter time frames from trigger to response. Emotions were evolved to allow flexible adaptations to important circumstances and challenges, and thus are subject to modification by learning and to cognitively-based adaptation (de Waal, 2019).

To prove VFF exists in a species, a workable approach is to demonstrate emotional valence variation from positive to negative in usage on different occasions of the same particular vocal type. Perhaps most important in order to demonstrate full VFF, it must be possible to demonstrate the occurrence of vocal events where there is *no discernible immediate function* – that is, the vocalization must be shown in some cases to be produced exploratorily and/or playfully. The peeps in Clay et al. (2015) were not shown to be produced exploratorily or playfully, and in fact no judgment was actually made about emotional valence (e.g., about facial expression, reaction of mother or other conspecifics, or other emotional indicators).

An additional problem with the study (Clay et al., 2015) was that it reported acoustic differentiation of the peeps occurring in the three contexts. If the data are correct, this acoustic demonstration undercuts the study’s expressed goal, and the data did not demonstrate the existence of a single vocal type (a peep) with three functions, but three types of peeps, each with its own function. That humans might call all these sounds peeps does not prove they were all of the same vocal type to the bonobos, and the acoustic data suggest they could have indeed consisted of three different types to the bonobos.

Although there has been no convincing demonstration to our knowledge of VFF in non-human primates, the issue remains open to further investigation. We propose that for vocalization to become an object of exploration, it is necessary for natural selection to tie vocal capacities to an emotional system engendering actions that do not necessarily produce immediate benefits. If Panksepp was right, this would be the Seeking system, present in all mammals. Vocal inclinations in humans appear to have been evolved to be connected to the Seeking system in much the same way exploratory actions with the hands appear to have been connected to this emotional system in primates generally.

## Barriers to Language Evolution: The Presumed Issue of Honesty

There must be barriers to language evolution, or we would not be the only creatures to have evolved it. The primary barrier that has been discussed in animal communication literature is based on the presumed competitive nature of signaling and its presumed resulting deceit. We are far from the first to express skepticism about this view or to outright reject it (see, e.g., Lachmann et al., 2001; Penn and Számadó, 2020). In Appendix A in the Supplementary Material, we address six key points that, in accord with our reasoning, counter the concerns and support the idea that the argument about deceit fails in providing an important barrier to either language evolution or stable communication in social-living non-humans. Here in the main text, we address what we believe to be the most fundamental reasons the idea of honest signaling as a barrier to language evolution is ill-conceived. These reasons are importantly related to the concept of VFF, as will be seen.

Consider the assumption that language is inherently honest. In fact, language is neither inherently honest nor inherently dishonest, a fact that can be illustrated with logical argument and examples alone. Acts of language are honest or dishonest depending on the circumstances they are intended to portray, and any mature speaker is capable of using language both ways. Perhaps the unsupportable claim that language needs to be honest is based on a confusion between the “meaning” of individual words, their semantics, and the way words are utilized to function (illocutionarily, see below) in communicative acts. The semantic meaning of a word, for example “rattlesnake,” is dependent, not on truth or falsity, but on an understanding among speakers of English that the word refers to a particular class of animals. The word is neither honest nor dishonest in and of itself. The bond between the word and its semantic content is a convention sustained by speakers of a language over long periods (often centuries), not an individual assertion that might be falsified. But if an English speaker, who knows the difference between pythons and rattlesnakes, intentionally asserts that a particular python is a rattlesnake, the speaker is lying. It is not the word that is the lie, but the use of it to label an animal incorrectly. The same person might of course use the word truthfully and correctly on a different occasion. Importantly, in language we can also say things that are meaningful but are neither true nor false – and we do it very often. For example, suppose one says: “Please remove the rattlesnake.” This could be a meaningful request; yet the request itself is neither true nor false. In writing the sentence about the rattlesnake, we have actually not made a request, but merely used a sentence as an example of a possible request. Nonetheless, the sentence, we have written uses meaningful English words in a meaningful and syntactically well-formed English sentence.

The fundamental misunderstanding that has been prevalent in animal communication literature based on the assumption that language is inherently honest can be unpacked and illuminated in the context of the Austinian distinction between illocutionary force and semantics (Austin, 1962). This distinction has been expanded in our own work so that it can apply not only to mature language, as it did for Austin, but also to human infant and animal communication (Oller, 2000; Griebel and Oller, 2008, 2014).

Illocutionary forces are the functions served in the here and now by communicative or potentially communicative acts. Illocutionary forces constitute the intentions that reflect underlying emotional/motivational states. Every production of a signal that has evolved to constitute a communication consists of at least one illocution, a performance of a communicative or potentially communicative act. For example, a scream emitted in Fear is an illocution, an “expression of Fear.” Human infant cry can be portrayed illocutionarily as an “expression of distress.” The hiss of a house cat can be viewed as a “threat.” These illocutionary acts are not words; they possess no semantics and do not refer to anything, but instead express a state and/or a communicative intention. They are performances inspired by the state or intention in the present, and consequently they are neither true nor false.

On the other hand, any semantic (or symbolic) act consists of *both a semantic reference and at least one illocution*. If one says “rattlesnake,” one may be performing a “labeling” illocution. Or with the same word, one might “correct” someone who had said “tree-root” (mistaking the snake for the root of a tree), and in so doing, one would produce two kinds of illocutionary functions in the same act, both a label and a correction. Saying “rattlesnake” could also be motivated by a fearful emotion, simultaneously invoking an “alarm” function along with the labeling function. Or one might say “rattlesnake” for the mere purpose of hearing the word, practicing it, or illustrating its pronunciation. Similarly, if one says “apple,” one might intend merely a “labeling” of a fruit hanging from a tree. On a different occasion, one might use the same word to “request” that an apple be handed over, simultaneously labeling and requesting. With any word or phrase, we can perform many different illocutions. We can label, request, confirm, deny, alert, stipulate, mock, question (seek information), criticize, practice pronunciation, and so on.

But semantic acts always involve something in addition to illocution; semantics also includes the transmission of information encoded in the content of what is said. This semantic content is both transmitted in the here and now and in a broader sense is detached from the here and now. The word “rattlesnake” is a semantic entity that refers in English on every occasion of usage to a particular class of animals regardless of the intended illocution. The semantic content is independent of space and time, every time the word is produced. The semantic tie between a word and its conceptual content exists even in the absence of its being spoken. We can think a word or phrase and thus invoke the appropriate concept. The concept is invoked also regardless of the affective valence of the illocutionary act, that is, whether we produce the word or phrase with negative, positive, or neutral affect (for example, fearfully, delightedly, or exploratorily), differences that tend to correspond to different classes of illocutions.

We have contended that natural animal signals are limited to illocutionary functions and do not transmit semantic information (Oller and Griebel, 2015). We have thus far found no convincing contradictory evidence – only animals extensively taught by humans have been shown to transmit semantic content (Griebel et al., 2016). Thus, each naturally occurring animal communicative action is a performance (as far as we know), a mapping in the here and now, from an emotional or bodily state to the signal that expresses it as an illocution. The action does not “say” or “assert” anything, and thus can have no truth value.

For example, during mating season, a red deer who lowers his larynx and produces a sound involving lower resonances than if the larynx had been left in its rest state (Fitch, 2000) performs an illocutionary act we might call “advertisement” or “showing off.” Did he lie by producing a sound with resonances suggesting a very large vocal tract and making himself sound larger than he is? No, because he did not say anything constituting an assertion that could be falsified. Male red deer in general produce mating calls with lowered larynges, and all of them do so for the same reason: the action makes them sound large and increases their probability of mating. Why? It appears female red deer choose to mate with males with deeper voices because deeper voices are related to greater body size and fitness. Any mutation that could have produced the inclination or capability to lower the larynx during mating calls could thus have been subject to runaway selection because it would have suggested great body size and fitness. The distinction between illocutionary force and semantics makes clear that illocutions are never true or false, because they are performances, not assertions. With semantic acts, however, we can indeed make claims about the world that may be subject to truth-value assessment.

Defenders of the idea that honest signaling is a barrier to language evolution might protest that they do not intend the term in animal communication theory to involve honesty as it can occur in language. Instead, they might argue that they only intend that the interaction between male and female deer involves the females being deceived into thinking the male deer they choose to mate with is bigger or more fit than he really is because he deceptively portrayed himself. This is an unnecessary conclusion. In the illocutionary interpretation, the male deer advertise by bellowing, and the female deer choose a mate on the basis of the effectiveness of the advertisement. Nothing can have been misinterpreted as true or false, because nothing was encoded semantically. Notice that all the advertising males lower their larynges. It is as if the honest signaling idea implies that all but one of them is lying. In that interpretation the females would have to be assumed to determine who is telling truth.

Propositions on the other hand (e.g., “there is a python” or “I am the biggest red deer in the forest”) are semantic and can indeed involve “assertions” bearing semantic content, which can (at least in many circumstances) be determined empirically to be true, false, or ambiguous as to truth value. To produce such a proposition, one must invoke symbolic elements (typically sentences composed of words) to encode it. A human male can potentially try to impress a female by claiming with words to be rich and famous, which can be proven to be objectively true or false. The mating bellow of the male red deer, on the other hand, cannot be proven to be true or false.

Because every linguistic proposition is free to express a vast array of possible illocutionary forces, there is always a complex mapping possible between any linguistic symbols and their possible illocutionary functions. Many-to-many mappings also obtain between linguistic symbols and the different emotions that can be expressed by them, since the emotions motivate and supply flavoring for the illocutions. “Rattlesnake” can be produced contemptuously or admiringly. It can even be produced with flat affect for no purpose other than to speak the word. Or it can by produced to educate, teaching the label. The options are seemingly endless.

Deceit is of course possible through propositions – it logically *has* to be possible in language given the requirement of functional flexibility – and consequently deceit is among the possible illocutions of any proposition. This is not a weakness of language but an aspect of its power. If and only if a communicative system has the power to transmit both illocutions and semantic contents, can truth and falsehood be assessed. Language also makes it possible to create imaginary worlds, where talk about those worlds can involve only imaginary truths and falsehoods. Literary and cinematic fiction involves purely imaginary communications that can be evaluated for truth only in the context of the imagination. Did Star Trek’s Captain Jean Luc Picard understand the Borg to be telling the truth, when it said “resistance is futile”? The question is not evaluable in the real world but is clearly meaningful and evaluable in the imaginary Star Trek world. The power of imagination supported by language yields vast possibilities in literature or cinema, but also in developing plans, providing explanations, coordinating actions, and so on.

In accord with our reasoning, the first step in selecting for such power, in moving beyond exclusively illocutionary communication, could not actually have involved selection for semantic capabilities. Rather a capability and inclination produced by selection had to form a foundation upon which a semantic system could later be built. This foundation, as we have argued above, involved the tendency in hominin infants to produce vocal fitness signals that had the (presumably unintentional) effect of revealing to caregivers their wellness and thus resulted in recurring nurturance of the infants through their long period of dependency. But crucially, the selection pressure was not on the quality of a *stereotyped* fitness signal, as in the case of the mating calls of the red deer, but on the tendency to explore various (not stereotyped) protophone types which could be interpreted as fitness signals. Selection for variety in fitness signaling can be found in mating and territorial calls in other species as well (e.g., birds and cetaceans), but to our knowledge these signals have never been shown to have VFF.

Importantly, one does not have to engage in fitness signaling intentionally in order for one’s vocalizations to be interpreted as fitness signals. One wonders how many animals and humans produce fitness advertisements without even being aware of what they are doing. Does a male bird sing out of joy or because he intentionally wants to impress a female or a rival? Are humans always aware of the display functions that are served by things they say or how they say them? It would appear that selection pressure has created positive reinforcement (pleasure and joy) for singing, dancing, or whatever behavioral display is the advertisement proving to be effective in various animal species.

The evidence suggests that from the perspective of the human infant in the first months of life, most of protophone production is not an attempt to signal anything, but rather to engage in exploration or vocal play (as inspired by the Seeking system), not directed to anyone. On other occasions, protophones do appear to be directed to a parent, in which case the vocalization might indeed represent an attempt on the infant part to bond with the parent – if not to signal fitness, at least to engage in social interaction in a playful way even in the first months of life (Gratier and Devouche, 2011; Dominguez et al., 2016; Yoo et al., 2018). From the caregiver perspective, infant vocalizations are presumably interpreted on many occasions in the same way the infant intends them, as explorations, for example. On other occasions protophones may be interpreted by caregivers as attempts to engage in social interaction. But at another level, the protophones heard by caregivers would seem always to supply fitness information, whether the infant intends them to supply such information or not. This result of protophone production is hypothesized in our approach to provide a basis for natural selection of infants (“parental selection” in the interpretation of Locke, 2006), who display their fitness through vocalizations, sometimes exploratory, sometimes interactive, sometimes emotionally expressive, but always in one way or another, providing rich information about infant state, well-being, and perhaps intelligence. To the extent that an infant vocalizes with the intention of “showing off,” it might be appropriate to say the illocutionary force does indeed involve “fitness signaling.” But judging infant intentions to this extent involves inferences that may be difficult to justify, just as it may be difficult to judge whether a bird intends his song to attract a mate or whether he merely intends to enjoy singing.

In summary, we see no barrier to the evolution of hominin vocal signaling because of an honesty issue. Language has to make both honest and dishonest communication possible, though a great many acts of language are not even evaluable with regard to honesty. The earliest communicative step away from the primate background in ancient hominis appears to have been the emergence of a capacity and an inclination to produce vocalizations as fitness signals long before there were words, and these fitness signals were neither true nor false.

## Possible Barriers To Evolution of VFF and Circumstances That May Have Helped Overcome Them

What barriers would have actually inhibited natural selection of vocal exploration and VFF? One possibility is that there may be advantages to relative silence in order to avoid alerting predators or competitors. While, we know of no systematic investigation to tie it down, a silence pressure seems obvious. Perhaps for primates in general, the value of vocalizing freely was simply not high enough to get it off the ground in the face of a countervailing pressure for silence. Our hypothesis for over a decade (Oller and Griebel, 2005, 2006; Griebel and Oller, 2008), also advocated by Locke (2006, 2009), has been that hominin evolution occurred in circumstances where the value of vocalizing flexibly exceeded that of the pressure for silence.

In particular, we have proposed that the altricial hominin infant was in need of long-term care (Locke and Bogin, 2006), and thus came under especially intense pressure to provide fitness signals that could influence caregivers to provide long-term nurturance and protection. Altriciality is assumed in this reasoning to have been at least partly a product of bipedalism and the consequent narrowing of the birth canal, the “obstetrical dilemma” that is believed to have caused a necessary reduction of fetal brain-case size in hominins (Wells et al., 2012; Gruss and Schmitt, 2015). This reduction is assumed to have been accomplished by natural selection to slow development in hominins, resulting in smaller brains at birth and more altricial bipedal hominins than their quadrupedal cousins, who did not face the same obstetrical challenge (Bogin, 1999). Greater altriciality resulted, according to the reasoning, in greater need for long-term care along with greater advantages to fitness signaling by the altricial young.

Altriciality may not have provided the only selective pressure on flexible vocalization by infants. There are relatively few cooperative breeders among the primates, with humans and callitrichids (a New World group including marmosets and tamarins) being the only ones that are well-documented as showing both extensive care and provisioning by “alloparents” (Hrdy and Burkart, 2020). Interestingly both these groups are highly vocal, and the callitrichids show signs of greater flexibility of vocalization than other primate species (Snowdon and Cleveland, 1984; Snowdon and Elowson, 1999; Snowdon, 2004; Zuberbühler, 2011; Burkart et al., 2018), although the issue of possible VFF has not been directly evaluated in them. The callitrichids may be the only non-human primate group that babbles (Elowson et al., 1998). It has been argued that cooperative breeding is a setting that implies special pressure on infants to signal their needs and their fitness to a wide variety of possible caregivers, the alloparents. Increased volubility of these signals, especially when utilized in optimal circumstances, could surely enhance the prospects for such infants. We propose that relative altriciality and cooperative breeding may have co-evolved, with both supplying selective pressure on vocal fitness signaling in the hominin case.

One might object that a vastly new vocalization capability involving VFF is not the only way to supply fitness information. Fitness information is supplied by many features of an infant: skin color or texture, breathing pattern, frequency of crying, responsivity to touch or voice, and so on. Our argument is that the altricial hominin infant, especially in its cooperative breeding environment, was under more intense pressure to supply fitness information than other apes because of the longer developmental period ahead, a period during which there was absolute need for caregiver sustenance and the greater variety of caregivers. This enhanced pressure seems to have produced a new human feature, one where infants could supply fitness information to caregivers who were occupied with other tasks (see Falk, 2004 for an argument that language evolution was influenced by the common requirement of “putting the baby down” during foraging), and this pressure may have been redoubled in the circumstance of cooperative breeding because there were many potential caregivers. The protophones produced by hominin infants regardless of circumstances seem to have supplied a near constant source of well-being information, allowing hominin caregivers to assess the information and select individual infants for enhanced investment.

We assume the same pressures due to altriciality in the distant past exist also for modern humans and have suggested possible lines of empirical test (see above) of the idea that fitness signaling by protophones is noticed by caregivers, who respond both physiologically and behaviorally. Furthermore, a comparative evaluation of caregiving in species varying in altriciality could be informative. We predict that the more altricial the newborns of the species are at birth, the more intense the caregiving will be and especially the more attentive the caregivers will be to signals of fitness. On cooperative breeding, one might imagine correlational research focused on groups where degree of alloparenting differs. The prediction would be that protophone volubility will be positively correlated with the extent of alloparenting across groups. An additional prediction would be that both parents and alloparents would be sensitive to recognizing and responding with care to fitness signals.

Another factor that may have played a role in the hominin vocal inclination is suggested by research suggesting ancient hominin groups became larger than other ape groups in very distant time (Dunbar, 1993, 1996). With larger groups the premium on silence may have been mitigated somewhat by safety in numbers, allowing ancient hominins to be more subject to selection pressure on vocal fitness signaling and more social signaling in general. Dunbar’s argument and that of Morris (1967) also emphasizes that as groups became larger, it became increasingly difficult to find enough time in the day to do all the grooming that primates seem to require to maintain peace in the group. Dunbar and Morris both proposed vocalization as having taken on a role similar to that of grooming in ancient hominins, because it was possible to vocally groom more efficiently, especially by including multiple recipients simultaneously.

An additional interpretation is that larger group sizes in hominins may have been in part made possible by emerging VFF. As vocalization became more frequent and more interactive, even if primarily between parents and infants, it surely would have been extended throughout the lifetime into utilization of vocalization to serve functions such as mating and alliance formation (vocal grooming). Such social vocalization usage may have fostered social cohesion with benefits not only to individuals but to groups of ancient hominins, whose numbers may have been able to expand in part because of the vocal connections and group commitments within their communities. In this interpretation, the vocal-grooming function may have co-evolved with the fitness-signaling function.

We are unaware of cross-species empirical research on this idea, but it could be tested for example, by evaluating the relative amounts of physical grooming and social vocalization in primate groups of varying sizes. Larger groups, other things being equal, might be expected to produce relatively larger amounts of social vocalization. Even in humans who live in hunter-gatherer societies, it may be possible to evaluate the relative amounts of physical grooming and social vocalization as a function of group size.

A critical feature of early hominin infant vocalization, selected as a fitness signal, according to our reasoning, was its connection with the Seeking system (see also Griebel and Oller, this volume), because it was this connection that motivated the copious production of protophones and gave them their flexibility. Vocalizations could be produced playfully without immediate utilitarian goals. The inclination to vocalize exploratorily appears to have been selected first and foremost as a form of investigation of the world, in this case the world of sound and its accompanying kinesthetics as produced by the vocal systems of the infants themselves. Assuming the vocal system is activated endogenously by the Seeking system, the activity can produce vocalizations varying in acoustic character for two reasons.

First, exploration can yield vocalizations that vary across a natural landscape of possible phonatory types corresponding to natural wells or “attractors” in a Waddingtonian landscape of vocal possibilities. Thus by self-organization, the exploration should produce variation and a tendency for categories to emerge. With increasing experience in exploration, the infant should learn to manipulate these categories, producing them repetitively and making them salient as categories. Indeed it has long been recognized that in modern human infants, several vocal categories tend to emerge in the first months: vowel-like sounds (vocants), squeals, growls, raspberries, and combinations of these (Zlatin-Laufer and Horii, 1977; Stark, 1978; Oller, 1981), and the repetition of each of these categories of sounds has been long recognized as a kind of vocal play, emerging at least by 5 months (Stark, 1980) but probably earlier (Jhang and Oller, 2017).

We have tested the identifiability of these protophone types auditorily (Oller et al., 2013) and based on human classification of spectrographic displays (Buder et al., 2008), and are currently comparing levels of agreement among human listeners compared with agreement between humans and automated acoustically-based identification. As a test of the extent to which exploration produces stable new sound types, we are currently involved in research on “clustering” of protophones of particular types (the tendency to produce particular types repetitively) across all-day recordings of typically developing infants and infants at risk for autism (Yoo et al., 2019b).

Second, acoustic properties characterizing particular emotional or affective states can modulate different protophone types so that each type, while maintaining acoustic signatures of its own, can simultaneously show acoustic variations tending to express differing emotional states. For example, a growl (which typically has harsh voice quality) might be produced on some occasions with no affective coloring but on other occasions with nasality and a whiny tone, along with a negative facial expression revealing discomfort. Intense discomfort might produce a louder version of the growl or an even more dysphonated and harsh version of the sound corresponding to a phonatory regime shift during at least part of the utterance. In a similar way, a vocant, which typically has normal phonation and reveals no affective positivity or negativity, might be produced with nasality and increased duration to signal distress (Yoo et al., 2019a). Squeals, which require high pitch, often in falsetto (or loft) register, can also be produced with affective neutrality, or can be colored by high intensity or the addition of intonational features suggesting distress.

We envision an evolutionary process where the tendency to vocalize flexibly in response to Seeking activation of the vocal system would have gradually become more frequent across (probably) millions of years. In successive generations, infants would have been increasingly fitness-signaling vocalizers, and the competition among infants for investment from caregivers would have persisted, a competition where fitness signaling vocalization would have always played a role (along with other fitness indicators, such as the appearance of skin health, coordination of movements, and so on). We also imagine that as the vocalizing infants grew up, they would have become more active users of vocalization in fitness signaling within their mature groups, with vocalization playing roles in mating and alliance formation.

Furthermore, as they became parents, the same individuals would have been sensitized to the value of vocalization as a signal of wellness, and they would have become increasingly attuned to noticing infant vocalizations as fitness signals. At some point, caregivers would have begun to elicit vocalizations by face-to-face vocal interaction, trying to gain access to information about fitness. Such face-to-face vocal interaction is common in human parents and infants (Brazelton et al., 1974; Cohn and Tronick, 1988; Jaffe et al., 2001; Hsu and Fogel, 2003), while never having been observed in other apes (Papoušek and Papoušek, 1983; Oller et al., 2019b). Not only would the endogenously-produced infant vocalizations have been useful indicators potentially benefiting infants who produced them, they would also have benefited their caregivers by providing a basis for allocating their investment energies. Thus fitness signaling was in the interest of both the infants and the caregivers. To the extent that there was competition, it was not primarily between caregiver and infant, but among infants who competed against each other for investment. A possible test of the competition among infants might be pursued in cases of multiple births. For example, one might seek to determine whether twins compete vocally in the sense that increases in protophone production by one twin produce increases in the other, independent of quotidian variations of production by each twin.

Another barrier to the evolution of vocal activity in the non-human primates seems likely attributable to relative lack of voluntary vocal control. The conclusion that voluntary vocalization is difficult for non-human primates has been noted in attempts to teach human-reared apes to produce anything resembling words – only the most minimal vocal “word learning” has been reported (e.g., Hayes and Hayes, 1951; Gardner et al., 1989). Similarly operant conditioning or social learning of vocalization in non-human primates has been shown to be difficult at best, with most authors emphasizing success in the realm of voiceless sounds, such as raspberries, smacking sounds, whistles, or whispered sounds (e.g., Marshall et al., 1999; Wich et al., 2009), with only minimal reported experience-driven modifications or modified uses of phonated vocalizations, and those modifications have applied to vocal types already existing in the relevant species repertoire (Sutton, 1979; Janik and Slater, 2000). The limits appear to be so severe that natural vocal learning in wild primates continues to be treated with a question mark about likely learning rather than an unambiguous positive conclusion (see, e.g., Crockford et al., 2004). A broad recent review concluded that the great bulk of vocal adjustments in non-human primates pertained to “vocal accommodation,” involving adjustments to existing call structure based on environmental noise or conspecific vocalizations, without primates’ learning to produce new sound types (Ruch et al., 2018).

In sharp contrast, a wide variety of other animals show clear vocal control and vocal learning, much more flexibly and easily achieved in the wild as well as in laboratory experiments. It appears that the vast majority of these animals either fly (e.g., songbirds, hummingbirds, parrots, and bats), have aquatic lifestyles (e.g., pinnipeds, dolphins, and whales), or have a history of aquatic or semi-aquatic lifestyles (e.g., elephants). In the case of the mammals that show impressive voluntary vocal control and vocal learning, most forage heavily or entirely in water, and must control their respiratory apparatus in such a way as to guage the amount of time necessary under water for each dive. This requirement imposes a necessity for voluntary control of the glottis (or other valve that manages respiratory flow). Since the glottis in primates is the apparatus that modulates respiration to create phonation, we propose that voluntary vocal control may have been facilitated by selection pressure on the voluntary control of the glottis that was naturally selected in hominins as a requirement of foraging by swimming and diving.

A hypothesis that ancient hominins lived at waterside, fishing and foraging in water by both wading and diving, and were heavily affected by selection pressures associated with these activities, appears to be gaining traction (Tobias, 2011; Attenborough, 2016). The idea has been on the table for many decades (Westenhoefer, 1942; Hardy, 1960; Morgan, 1997), but has been opposed by most of the community of paleoanthropology (e.g., Langdon, 1997), which is still primarily committed to the savannah hypothesis of human origins. Yet savannah living offers no integrated solution to explain the suite of characteristics that mark humans as remarkably distinct from their primate cousins, most importantly bipedalism, hairlessness, extensive subcutaneous fat, and voluntary phonatory control (for additional features and elaborations see, e.g., Niemitz, 2010; Gislen and Schagatay, 2011; Schagatay, 2011; Verhaegen et al., 2011). The idea is that ancient apes (perhaps the ancestors of both greater and lesser apes) spent significant periods of their evolution foraging in water, a pattern that may have influenced the evolution of preferential bipedalism on land (in hominins and gibbons) or other forms of special ambulation such as knuckle walking (in, e.g., chimpanzees and bonobos). In accord with reasoning by some supporters of this “waterside hypothesis,” the hominins may have been the apes that stayed the longest in waterside living, experiencing to a much greater extent than other apes the special selection pressures of wading, swimming, and diving to forage. In accord with the hypothesis, hominins were evolved to be fully bipedal because wading places strong selection pressures on upright gait (see Kuliukas et al., 2009) and to possess the additional characteristics common in marine (and previously marine) mammals, as listed above.

This idea presents a possible basis upon which selection pressure on vocal fitness signaling could have taken hold in ancient hominin infants more easily than in other primates. If they were preadapted for more voluntary glottal control, they may have as a consequence been more susceptible to selection pressures on voluntary phonation than infants of other primate species. To our knowledge there has been no systematic research correlating the amount of diving done by various species with degree of vocal control. Such work should take into account the lifestyles of the species, since solitary creatures should not be expected to be as inclined to use social vocalization as gregarious species. Mating patterns should also be considered because mating songs also require vocal learning. Another test of the possible influence of hominin waterside living on vocal control could involve experimental studies of breath holding among primates. Instrumental conditioning research could conceivably make it possible to determine the degree to which apes and other primates can be taught to hold their breath.

The thoughts expressed here about possible impediments to the evolution of language are surely incomplete. Yet speculations and creative research on possible forces both favoring and inhibiting evolution of vocal flexibility hold promise in illuminating the origins of language.

## Data Availability Statement

The original contributions presented in the study are included in the article/Supplementary Material; further inquiries can be directed to the corresponding author.

## Author Contributions

DKO and UG wrote the paper and conceived of the theoretical framework it expresses. Both authors contributed to the article and approved the submitted version.

### Conflict of Interest

The authors declare that the research was conducted in the absence of any commercial or financial relationships that could be construed as a potential conflict of interest.
