# Supplementary material for: Functionally Flexible Signaling and the Origin of Language
Source: Front Psychol. 2021 Jan 26;11:626138. doi: 10.3389/fpsyg.2020.626138 (PMC7870697; doi:10.3389/fpsyg.2020.626138)
Supplement: Supplementary file 1 [file Data_Sheet_1.docx]

**Functionally flexible signaling and the origin of language**

Oller and Griebel

Appendix A

**Barriers to language evolution: The presumed problems of deceit and honesty**

There must be barriers to language evolution, or we would not be the only creatures to have evolved it. One important possible barrier that has been discussed in animal communication literature is based on the presumed competitive nature of signaling and its presumed resulting deceit.

The idea that animal communication is a Darwinian contest between manipulative senders of communications and receivers who attempt to limit the manipulation is consistent with the notion of the selfish gene (Dawkins, 1976) and, by extension, with the self-interested nature of actions by any organism, the holder and purveyor of genes. Assuming inherent selfishness of senders, the leading proposal in the existing biological literature to make “honest” communication possible is to make it costly. An arguably inevitable implication of the proposal is that reliable communication must be inherently wasteful. Knight (2016) characterizes this oddity in animal communication, according to Zahavi (1997), as a major blockade against language evolution.

Another feature of Zahavi’s reasoning is that communication in animals is necessarily analog in nature, not including the discreteness of phonemes and words in human languages. This reasoning is consistent with the idea that the expression of emotion naturally involves a continuum of intensity; the continuum is of use to senders in expressing their states and to receivers in understanding them. Of course, language does not prevent the analog expression of state intensities. In fact, any word or sentence, in accord with the functional flexibility principle of language discussed in the main text, can be expressed at any degree of emotional intensity and can express any kind of emotion. A key difference between human language and most animal communication is that words and sentences *also* express semantic content that is inherently free of emotional valence and wherein intensity is irrelevant. A seeming paradox: Words inherently possess emotionally detached semantic content, but they can, on any occasion of utterance, express any degree or type of emotion. For example, the word “apple” can be expressed on two different occasions with delight or derision, invoking two opposite functional valences, but in both cases the semantically encoded concept of the class of apples is also invoked. This is a level of flexibility that is not possible in animal communication as far as is known. Zahavi’s reasoning ignores these fundamental issues which are at the basis for the primary arguments against his reasoning in the main text.

Others have also expressed deep skepticism about the contention that honesty poses a barrier to communicative evolution and especially to language evolution (Lachmann, Számadó, & Bergstrom, 2001; Maynard Smith & Harper, 2003; Penn & Számadó, 2020). Here we address six key points, that, in accord with our reasoning, counter the concerns and support the idea that the Zahavian argument about deceit fails in providing an important barrier to either language evolution or stable communication in social-living non-human animals.

1. Deceit in animal communication is hypothetical; its very existence is hard to prove. If it occurs at all—we are inclined to believe it does—it is naturally limited to very special and infrequent cases. Consider a call suggesting Fear, a call that could normally also induce Fear in conspecifics. The conspecifics might read the call as having been inspired by the caller’s having seen a predator. But what if the call was emitted, even without the sender’s having perceived a predator, as an intentional attempt to make the conspecifics run away from a food source that had just been discovered? If a Fear signal is produced as described, it must be subject to degradation as an alarm for conspecifics (they would come to recognize the deceit in “crying wolf” too often), and indeed this sort of deception would constitute a challenge for maintenance of the Fear call as an alarm. Maynard-Smith and Harper (2003) and others have argued that deception must be rare in any evolved group communication system precisely because the value of signals would degrade to the point of uselessness otherwise.

Communication in highly social groups such as primates would seem, then, to involve a strong limitation on deceit of this sort. Indeed, evidence of such deceit is uniformly anecdotal and subject to other interpretations not invoking deceit, e.g. a sender can be simply mistaken in assuming a predator is present. We do not deny the existence of such deceit, but advocate, agreeing with Maynard-Smith and Harper, that it must be rare. Especially in highly social animals, the necessity of group cohesion requires signals to be on balance clearly interpretable, preventing deceivers from having their way because members of a tight-knit group know each other well, and they have reputations to uphold.

1. In stark contrast to the literature’s worries about self-interest overwhelming animal communication evolution, we agree with Maynard-Smith and Harper in the contention that stable signals in any animal society must in fact *benefit* *both* *senders and receivers* (let’s call this the mutual benefit principle) in the long run, otherwise no signaling system would evolve in the first place. Consider the fact that vocal threats are not merely to the benefit of the producer. If understood by the receiver, they may supply a reason to submit rather than fighting because they may accurately display the emotional state of the producer and therefore the accompanying motivational state of the threatening animal. Threats and counter threats may result in one party backing down, and if nobody gets hurt, the communications will have benefited both parties. In fact, vocal threats very likely evolved exactly because they provide a means of resolving disputes without injury, just as in the case of displays of morphological signals in ritualized fights (involving for example displays of weaponry such as antlers, baring of teeth, and so on).

Other signals, including vocalizations, can also be seen generally to benefit both sender and receiver: e.g., contact calls are inherently cooperative, and infant isolation calls have the potential effect of reuniting parent and offspring. Primate parents are known to call their infants if they are separated, surely benefiting both parties. Submission signals can be viewed as advantageous because they prevent unnecessary attack and potential injury by acknowledging and/or reinforcing a dominance hierarchy. Primate communication systems appear to be largely stable because they require that all parties use and understand most communicative acts in ways that are consistent for the roles of the senders and for the functions that are served.

1. While primate life has often been portrayed with a primary emphasis on conflict and aggression, in fact the great bulk of primate life is conducted without observed aggression. A variety of observational research reviewed by Sussman and Garber (2004) provides evidence that in primate species that have been evaluated, agonistic interactions are outnumbered by affiliative and cooperative interactions by a factor as large as 50. The authors argue that the primary driving force behind sociality in primates is not competition, but mutual benefits and collective advantages that obtain within a well-functioning social unit.

Nowak and Highfield (2011) have argued that in spite of the Darwinian emphasis on survival of the fittest and the implication that competition drives evolution, cooperation is far more fundamental in life than has previously been recognized. Based heavily on mathematical modeling, the authors emphasize that cooperation is the engine of innovation and the foundation for success in group-living species. Humans, in accord with their reasoning, are SuperCooperators, who have supremely exploited the advantages of cooperation, especially to the benefit of individuals within groups who show high degrees of cooperativeness. The modeling and reasoning support the idea that cooperative and even altruistic behavior is valuable enough to form a basis for positive selection on cooperation, and by implication to form a basis for selection on communicative signals that have mutual benefits for communicative participants in social groups.

1. Most animal signaling does not involve the communicative issues that are the primary focus of Zahavi’s reasoning. The widespread idea that reliable signals have to be costly has merit in explaining ornaments or other costly signals that play a role in fitness advertisements in mating or territorial defense for a variety of species (even in cases of monogamy, see Hooper and Miller, 2008). But most communication is not directed at fitness advertising for mating or territorial defense and most involves no costly bodily or vocal ornaments/handicaps. The Zahavian reasoning seems to assume that communication involves primarily conflict between senders and receivers, but the mutual benefit principle emphasizes other communicative functions and clarifies the fact that even in cases of conflict, an equilibrium can be established, making both aggression and submission signals useful to both sender and receiver.

The Zahavian principle is based on observations of animals that do not live primarily in social groups resembling those of many mammals and most primates. Costly ornaments and costly sexual displays pertain primarily to animals whose social lives are typically limited to lekking for mate selection, sometimes followed by pair-based rearing of young. Focus on such species appears to have biased Zahavian reasoning to ignore the vastly more complex and largely cooperative communicative relations that occur in complex social groups such as those seen in most primates, where group living is the rule.

1. If our reasoning is on target, the deepest foundations of language were evolved principally in the context of communication between mothers and infants and would thus have been supported by the strongest form of kin selection, where both parties have common interests that limit manipulation and evolve by transmission of information that overwhelmingly tends to benefit both parties (see review in Fitch, 2004). Kin communication is often viewed as a key to cooperative communication since altruism can be based on shared genetic interests (Hamilton, 1963). In social groups such as those of most primates, there is extensive kin relationship at stake and consequently extensive basis for cooperative communication. We might add that primates know the members of their group individually and remember prior interactions—this awareness provides a basis for tit-for-tat altruism—an additional source of cooperative behaviors.
2. The principle of “comparative advantage”, derived from economic theory of the early 19^th^ century (Bernhofen & Brown, 2018; Ricardo, 1817/1966; Ruffin, 2002), is worth noting in the context of the concerns that self-interest may overwhelm the potential evolution of language. Comparative advantage has been referred to as “Ricardo’s magic trick” (Ridley, 2010). In short, the thought experiment created by Ricardo is a proof that trade can be beneficial to both parties even in cases where both think they are getting the better deal or even cheating the other. The argument has been taken as key evidence that there is a practical way to minimize international conflict—"trade with each other instead of making war”—which may be seen as analogous to “communicate instead of fighting”. The thought experiment illustrates how both parties can profit from trade even in counterintuitive circumstances where both parties produce goods the other could produce more efficiently by themselves. Specialization of production across groups that trade creates unanticipated benefits for both. The “comparative advantage” is unexpected but clearly present. It should perhaps be no surprise, then, that much that is communicated between primates is also advantageous to both parties, regardless of the operation of self-interest on both sides.

Appendix B

**Foundations for language in non-human primate communication**

Our proposal contrasts with many prior suggestions about possible routes toward language in hominin history. Proposals by linguists and cognitive scientists tend to offer accounts of how advanced properties of language such as semantics and syntax could have arisen (Bickerton, 1990; Cangelosi & Parisi, 2002; Chater, Reali, & Christiansen, 2009; Pinker, 1994). We view such efforts as incomplete because they do not address the cognitive and infraphonological foundations that would have to have been evolved to make semantics and syntax possible.

Our proposal is aimed at the “preadaptations” (Christiansen & Dale, 2004; Christiansen & Kirby, 2003) that would have been required, and that approach makes it possible to address the very first steps of evolution that would have moved hominins in a direction ultimately compatible with language. Much of the literature in primatology that has attempted to address foundations for language that might be observable in non-human primates has been caught up in an attempt to leap past these necessary preadaptations, seeking capabilities for phonology (Andrew, 1976), semantics (Seyfarth & Cheney, 2010; Seyfarth, Cheney, & Marler, 1980), and even syntax (Arnold & Zuberbühler, 2006; Clay & Zuberbühler, 2011), seemingly trying to prove these capacities are already present in primitive forms in non-human primates. This is not the place to explain in detail the many reasons we view these claims to be misguided and to represent overinterpretations of empirical findings. The key point is simply that vocal functional flexibility is required as a prior foundation for all of these capabilities as discussed extensively in the main text.

A more productive question to consider than whether non-human primates have phonology, semantics, or syntax is the extent to which they show foundational capacities from which phonology, semantics, or syntax might develop. One fruitful realm of investigation is babbling-like sounds. Marmoset monkeys (both pygmy marmosets and common marmosets) appear to produce variable sounds in infancy that appear indeed to be free of immediate functions at least in some cases (Elowson, Snowdon, & Lazaro-Perea, 1998; Snowdon & Cleveland, 1984; Takahashi et al., 2015; Takahashi, Narayanan, & Ghazanfar, 2013). Are they functionally flexible? So far it is not clear, but the fact that cooperative breeding is involved hints at possible selection pressures on fitness signaling. Furthermore, the sounds of marmoset babbling have been shown to be involved in parent infant turn-taking, an obvious additional foundation for language. Since we know of no evidence of such babbling in apes, it may be that convergent evolution of relatively free infant vocalizations under the pressure of parental selection has created an analogous capability in the hominin line and in this distantly related group of New World monkeys. Indeed both altriciality and cooperative breeding may have supported natural selection of vocal flexibility in ancient hominins and may continue to support it currently.

Another realm of primate vocal communication that has been suggested as a particularly hopeful one in providing a foundation for language evolution concerns grooming vocalizations and lip smacks (Blount, 1985; Locke, 2008; Whitham, Gerald, & Maestripieri, 2007), sounds that suggest social connectivity of pairs of individuals. We wonder if it is possible that the inclination to produce such socially-directed sounds, produced in cooperative circumstances with apparently low intensity emotion, could have been co-opted in the hominin line by selection pressure for flexibility of the production of such sounds, presumably through evolution of an association with the Seeking system (Panksepp, 2011; Panksepp & Biven, 2012). Contact calls emitted in various species of primates are, like grooming vocalizations, also socially cooperative. These also may be generated by mechanisms that involve relatively low intensity emotions, and it is tempting to speculate that whatever those mechanisms are, they may have been possible to modify through evolution in ways that may have formed foundations for more flexible sound production.

**References**

Andrew, R. J. (1976). Use of formants in the grunts of baboons and other nonhuman primates. In S. Harnad, H. Steklis, & J. Lancaster (Eds.), *The origins and evolution of language* (Vol. 280).

Arnold, K., & Zuberbühler, K. (2006). Semantic combinations in primate calls. *Nature, 441*, 303. doi:doi: 10.1038/441303a

Bernhofen, D. M., & Brown, J. C. (2018). On the Genius Behind David Ricardo’s 1817 Formulation of Comparative Advantage. *Journal of Economic Perspectives, 32*(4), 227–240.

Bickerton, D. (1990). *Language and Species*. Chicago: University of Chicago Press.

Blount, B. G. (1985). "Girney" vocalizations among Japanese macaque females: Context and function. *Primates, 26*(4), 424-435.

Cangelosi, A., & Parisi, D. (2002). Computer simulation: A new scientific approach to study of language evolution. In A. Cangelosi & D. Parisi (Eds.), *Simulating language evolution* (pp. 3-28). London: Springer-Verlag.

Chater, N., Reali, F., & Christiansen, M. H. (2009). Restrictions on biological adaptation in language evolution. *Proceedings of the National Academy of Sciences, 106*, 1015-1020. doi:10.1073/pnas.0807191106

Christiansen, M. H., & Dale, R. (2004). The role of learning and development in language evolution: A connectionist perspective. In D. K. Oller & U. Griebel (Eds.), *The Evolution of Communication Systems: A Comparative Approach*: MIT Press.

Christiansen, M. H., & Kirby, S. (2003). Language evolution: Consensus and controversies. *Trends in Cognitive Sciences, 7*(7), 300-307.

Clay, Z., & Zuberbühler, K. (2011). Bonobos Extract Meaning from Call Sequences. *PLoS One, 6*(4), 1-10.

Dawkins, R. (1976). *The selfish gene*. Oxford: Oxford University Press.

Elowson, A. M., Snowdon, C. T., & Lazaro-Perea, C. (1998). 'Babbling' and social context in infant monkeys: parallels to human infants. *Trends in Cognitive Sciences, 2*(1), 31-37.

Fitch, W. T. (2004). Evolving honest communication systems: kin selection and "mother tongues". In D. K. Oller & U. Griebel (Eds.), *The Evolution of Communication Systems: A Comparative Approach* (pp. 275-296). Cambridge, MA: MIT Press.

Hamilton, W. D. (1963). The evolution of altruistic behavior. *American Naturalist, 97*, 354-356.

Hooper, P. L., & Miller, G. F. (2008). Mutual Mate Choice Can Drive Costly Signaling Even Under Perfect Monogamy. *Adaptive Behavior, 16*(1), 53-70. doi:10.1177/1059712307087283

Knight, C. (2016). Puzzles and mysteries in the origins of language. *Language & Communication, 50*, 12-21. doi:<https://doi.org/10.1016/j.langcom.2016.09.002>

Lachmann, M., Számadó, S., & Bergstrom, C. T. (2001). Cost and conflict in animal signals and human language. *Proceedings of the National Academy of Sciences, 98*(23), 13189-13194. doi:10.1073/pnas.231216498

Locke, J. L. (2008). Lipsmacking and babbling: Syllables, Sociality, and Survival. In B. L. Davis & K. Zajdo (Eds.), *The Syllable in Speech Production* (pp. 111-132). New York: Erlbaum.

Maynard Smith, J., & Harper, D. (2003). *Animal Signals*. Oxford: Oxford University Press.

Nowak, M. A., & Highfield, R. (2011). *SuperCooperators: Altruism, Evolution, and Why We Need Each Other to Succeed*. New York: Free Press.

Panksepp, J. (2011). Toward a cross-species neuroscientific understanding of the affective mind: do animals have emotional feelings? *American Journal of Primatology, 73*(6), 545-561.

Panksepp, J., & Biven, L. (2012). *The Archaeology of Mind: Neuroevolutionary Origins of Human Emotion*. New York: W. W. Norton & Company.

Penn, D. J., & Számadó, S. (2020). The Handicap Principle: how an erroneous hypothesis became a scientific principle. *Biological Reviews, 95*(1), 267-290. doi:10.1111/brv.12563

Pinker, S. (1994). *The language instinct*. New York: Harper Perennial.

Ricardo, D. (1817/1966). *On the Principles of Political Economy and Taxation: Reprinted in The Works and Correspondence of David Ricardo* (P. Sraffa. Ed.). London: Cambridge University Press.

Ridley, M. (2010). *The Rational Optimist: How Prosperity Evolves*. New York: Harper-Collins.

Ruffin, R. J. (2002). David Ricardo’s Discovery of Comparative Advantage. *History of Political Economy, 34*, 727-748.

Seyfarth, R. M., & Cheney, D. L. (2010). Production, usage, and comprehension in animal vocalizations. *Brain and Language, 115*, 92-100.

Seyfarth, R. M., Cheney, D. L., & Marler, P. (1980). Vervet monkey alarm calls: Semantic communication in a free-ranging primate. *Animal Behaviour, 28*, 1070-1094.

Snowdon, C. T., & Cleveland, J. (1984). "Conversations" among Pygmy marmosets. *American Journal of Primatology, 7*, 15-20.

Sussman, R. W., & Garber, P. A. (2004). Rethinking sociality: Cooperation and aggression among primates. In R. W. Sussman & A. R. Chapman (Eds.), *The Origins and Nature of Sociality* (pp. 161-190). Hawthorne, NY: Aldine de Gruyter.

Takahashi, D. Y., Fenley, A. R., Teramoto, Y., Narayanan, D. Z., Borjon, J. I., Holmes, P., & Ghazanfar, A. A. (2015). The developmental dynamics of marmoset monkey vocal production. *Science, 349*(6249), 734-738.

Takahashi, D. Y., Narayanan, D. Z., & Ghazanfar, A. A. (2013). Coupled Oscillator Dynamics of Vocal Turn-Taking in Monkeys. *Current Biology, 23*, 2162–2168. doi:doi.org/10.1016/j.cub.2013.09.005

Whitham, J. C., Gerald, M. S., & Maestripieri, D. (2007). Intended Receivers and Functional Significance of Grunt and Girney Vocalizations in Free-Ranging Female Rhesus Macaques. *Ethology, 113*(9), 862–874. doi:doi:10.1111/j.1439-0310.2007.01381.x

Zahavi, A., & Zahavi, A. (1997). *The Handicap Principle. A Missing Piece in Darwin’s Puzzle*. Oxford, UK: Oxford University Press.
